# Stent-assisted coiling using the pEGASUS-HPC stent for acutely ruptured wide-necked intracranial aneurysms: A multicenter retrospective study

**DOI:** 10.1177/15910199251357993

**Published:** 2025-07-13

**Authors:** Abdallah Aburub, Zakarya Ali, Ali Khanafer, Tawfik Moher Alsady, Oussama Dob, Christopher Nimsky, Alexander Grote, Bayan Alhaj Mouatafa, Julia Korthäuer, Stephan Felber, Hans Henkes, André Kemmling, Mohammad Almohammad

**Affiliations:** 1 Department of Diagnostic and Interventional Neuroradiology, University Hospital Marburg, Philipps-University Marburg, Marburg, Germany; 2 Central Institute for Diagnostic and Interventional Radiology, Neuroradiology, and Nuclear Medicine, Sana Klinikum Offenbach, Offenbach, Germany; 3 Department of Neuroradiology, Klinikum Stuttgart – Katharinenhospital, Stuttgart, Germany; 4 Department of Neuroradiology, Rhön Klinikum, Campus Bad Neustadt, Bad Neustadt an der Saale, Koblenz, Germany; 5 Department of Neurosurgery, University Hospital Marburg, Philipps-University Marburg, Marburg, Germany; 6 Institute for Diagnostic and Interventional Radiology and Neuroradiology, Stiftungsklinikum Mittelrhein Koblenz, Marburg, Germany

**Keywords:** pEGASUS-HPC, stent, coiling, aneurysm, subarachnoid hemorrhage

## Abstract

**Background:**

Stent-assisted coiling (SAC) is increasingly used to treat ruptured wide-necked intracranial aneurysms. The pEGASUS-HPC stent, featuring a hydrophilic polymer coating (HPC) to reduce thrombogenicity, may offer a safe option in subarachnoid hemorrhage (SAH). This study evaluates its safety and efficacy in a multicenter retrospective cohort.

**Methods:**

Between July 2021 and June 2024, 22 patients with ruptured wide-necked aneurysms were treated with pEGASUS-HPC SAC at four neurovascular centers. Procedural success, aneurysm occlusion (Modified Raymond-Roy Classification, MRRC), and clinical outcomes based on the modified Rankin Scale (mRS) at discharge and follow-up were assessed. Complications and mortality were analyzed in relation to clinical and procedural factors.

**Results:**

Stent implantation and coil embolization were successfully performed in all patients. Immediate complete occlusion (MRRC I) was achieved in 19 cases (86.4%) and reached 100% at the 3-month follow-up. The median mRS improved from 3 (2–5) at discharge to 0 (0–2) at 3 months and 0 (0–1) at final follow-up, indicating sustained recovery. In 27.3% of cases, Y-stenting was required, reflecting bifurcation complexity. One patient (4.6%) had transient in-stent thrombosis, which resolved with tirofiban. All complications were minor and managed conservatively. No retreatment was required. All three deaths (13.6%) occurred in patients with Hunt and Hess grade V and basilar artery aneurysms.

**Conclusion:**

The pEGASUS-HPC stent showed a favorable safety profile with high occlusion and recovery rates in ruptured wide-necked aneurysms. These findings support the use of surface-modified stents in the acute setting and highlight the need for prospective studies to confirm long-term safety and efficacy.

## Introduction

Intracranial aneurysms, characterized by localized dilations of cerebral arteries, pose significant risks due to their potential for rupture, leading to subarachnoid hemorrhage (SAH) and substantial morbidity and mortality.^1^ Wide-necked aneurysms pose significant therapeutic challenges. Endovascular coiling has become a key minimally invasive alternative to surgical clipping, especially after the International Subarachnoid Aneurysm Trial (ISAT) demonstrated favorable outcomes in selected cases.^2–5^ However, wide-necked aneurysms often require adjunctive techniques to prevent coil prolapse into the parent vessel. Stent-assisted coiling (SAC) is widely used in these cases, offering a scaffold to secure coil placement and promote endothelialization across the aneurysm neck.^6,7^ Despite its benefits, SAC in acutely ruptured aneurysms is used cautiously due to the need for dual antiplatelet therapy, which may heighten the risk of hemorrhagic complications during the acute phase of SAH.^8,9^

The pEGASUS-HPC stent, developed by Phenox GmbH (Bochum, Germany), is a low-profile, self-expanding, open-cell, laser-cut device designed for the treatment of wide-neck aneurysms, arterial dissections, and intracranial stenosis.^10,11^ Its flexible design allows optimal adaptation to various vessel anatomies, and it can be delivered through standard 0.0165″ inner diameter microcatheters used for coiling, eliminating the need for microcatheter exchange. The stent also features also a hydrophilic polymer coating (HPC) that reduces thrombogenicity by inhibiting platelet adhesion on the stent surface. This HPC modification could be particularly advantageous in the setting of acute SAH, as it reduces the need for dual antiplatelet therapy and thereby reduces the risk of hemorrhagic complications.^12,13^

Initial clinical experience with the pEGASUS-HPC stent has been encouraging. A multicenter retrospective case series reported successful stent deployment in all cases, high immediate occlusion rates, and low periprocedural complication rates.^10^ Another study emphasized the ease of use of the pEGASUS-HPC stent and its potential to broaden the spectrum of treatable aneurysms while reducing the need for dual antiplatelet therapy.^14^

Despite promising initial reports on the feasibility and technical performance of the pEGASUS-HPC stent—such as those by Boxberg et al. and Pielenz et al.^11,14^—its specific use in acutely ruptured wide-necked intracranial aneurysms remains insufficiently studied. Previous studies have focused primarily on unruptured or mixed-case cohorts, with limited insight into the acute SAH setting, where antiplatelet management and procedural timing are critical. The primary objective of this study was to evaluate the safety, feasibility, and efficacy of the pEGASUS-HPC stent in SAC of ruptured wide-necked aneurysms, based on clinical and radiological outcomes in a multicenter cohort treated under a standardized antiplatelet protocol.

## Methods

### Study design

This retrospective multicenter study was conducted at four neurovascular centers, with consecutive data screened between June 2021 and June 2024. Ethical approval was granted by the local Ethics Committee (IRB: 24-180 RS); informed consent was waived due to the retrospective design and anonymized data collection and analysis.

### Study population

Inclusion criteria: patients were eligible for inclusion if they met all of the following criteria:

(a) Presentation with acutely ruptured intracranial aneurysm. (b) Aneurysm morphology classified as saccular or dissecting. (c) Presence of a wide-necked configuration, defined—as in previous studies—as a neck diameter ≥ 4 mm or a dome-to-neck ratio < 2.^15^ (d) Treatment with SAC using exclusively the pEGASUS-HPC stent; no other stent systems were used during the study period.

The only exclusion criterion was aneurysm morphology classified as fusiform or blister-like, as these were primarily managed using alternative techniques, particularly flow diverter systems. No patients were excluded for other reasons.

Y-stenting was employed in anatomically complex bifurcation aneurysms where single-stent coverage of the aneurysm neck was not feasible due to the wide angulation or geometry of the involved branches.

### Study procedures

All procedures were performed under general anesthesia using a biplane angiographic system. Vascular access was obtained via an 8 French short femoral sheath. A triaxial access system was advanced under 3D rotational angiography guidance and consisted of a 6 French NeuronMAX 088 long sheath (Penumbra), a 5 French Sofia distal access catheter (MicroVention), and an Excelsior SL-10 microcatheter (0.0165″ × 150 cm, Stryker). The pEGASUS-HPC stent was delivered through the Excelsior SL-10 microcatheter, which was subsequently used for coil embolization of the aneurysm using detachable coils (Target, Stryker). At the beginning of the procedure, all patients received an intravenous bolus of 4000 IU heparin.

To minimize confounding bias, all procedures were conducted using a uniform SAC technique and a standardized antiplatelet regimen based on predefined protocols applied across participating centers. Following stent deployment, a weight-adjusted intravenous bolus of tirofiban was administered. Simultaneously, the aneurysm sac was catheterized via the microcatheter, and coil embolization was performed. A final angiographic run was carried out to confirm adequate aneurysm occlusion and to exclude any procedure-related complications. Upon completion of the procedure, a non-contrast cranial CT scan was performed to rule out hemorrhagic complications, followed by a loading dose of 60 mg prasugrel. Two hours after the intervention, a multimodal CT protocol—including CT angiography and CT perfusion imaging—was conducted to exclude in-stent thrombosis. Beginning on the following day, patients received 10 mg of prasugrel once daily for a period of three months. Platelet inhibition was monitored using the VerifyNow^®^ system (Werfen), and the prasugrel dose was adjusted accordingly. Thereafter, patients were switched to acetylsalicylic acid (ASA) 100 mg once daily, which was continued as lifelong secondary prophylaxis.

### Clinical endpoints

Clinical outcomes were evaluated based on changes in the modified Rankin Scale (mRS), assessed at discharge and at follow-ups at 3 months, 6 months, and 1 year. Procedural safety was additionally assessed by recording any procedure-related complications, including acute vessel occlusion, in-stent thrombosis, and aneurysm re-rupture.

### Imaging endpoints

The primary imaging endpoint was the degree of aneurysmal occlusion following pEGASUS-HPC SAC. Immediate procedural success was assessed using the Modified Raymond-Roy Classification (MRRC) based on post-procedural angiographic findings. Aneurysm occlusion was re-evaluated at 3 months with follow-up digital subtraction angiography (DSA). Further imaging at 6 months and 1 year was performed using magnetic resonance imaging (MRI) with time-of-flight (TOF) sequences, or computed tomography angiography (CTA) in cases of MRI contraindications.

### Data collection

Demographic, clinical, imaging, procedural, and follow-up data were retrospectively collected and managed in Microsoft Excel. Procedural information, including materials, techniques, and complications, was systematically documented. Follow-up imaging was evaluated via the institutional PACS. Data integrity was ensured through standardized documentation and cross-validation.

### Data analysis

Continuous variables were reported as means ± standard deviations or medians with interquartile ranges (IQRs), depending on data distribution. Categorical variables were summarized as frequencies and percentages. Group comparisons (e.g. survivors vs deceased) were conducted using t-tests or Mann–Whitney U tests for continuous data, and chi-square or Fisher's exact tests for categorical data. Functional outcomes (mRS scores), treated as ordinal variables, were evaluated at discharge and last follow-up using the Wilcoxon signed-rank test. Mean differences and 95% confidence intervals were calculated, and significance was determined using paired t-tests (*p* < 0.05). Analyses were based on complete case data without imputation and conducted using Jamovi version 27.0 (IBM Corp., Armonk, NY).

## Results

### Demographic, clinical and imaging characteristics

The study included 22 patients with a mean age of 63.4 ± 12.5 years; 15 patients (68.2%) were female. Aneurysms were more frequently located in the anterior circulation, affecting 13 patients (59.1%). The anterior communicating artery (AComA) and the basilar artery (BA) were the most common sites, each in 7 patients (31.8%). Accordingly, midline aneurysms (AComA or BA) were present in 14 patients (63.6%), while lateralized aneurysms were equally distributed between left and right, with 4 patients each (18.2%). Most aneurysms were saccular (21 patients, 95.4%), with only one dissecting aneurysm observed. No blister or fusiform aneurysms were treated with SAC. The average dome-to-neck ratio was 1.4 ± 1.0. At presentation, 13 patients (60.5%) had Hunt and Hess grades III–V. According to the Fisher scale, grade IV was the most common finding, observed in 8 patients (36.4%). Baseline characteristics are summarized in Table 1.

**Table 1.** Demographic, clinical, and imaging characteristics.

Parameters		*N* (%)/mean ± SD
Age		63.4 ± 12.5
Gender	Female	15 (68.2%)
Aneurysm location	AComA	7 (31.8%)
	BA	7 (31.8%)
	PComA	3 (13.6%)
	MCA	2 (9.1%)
	PICA	2 (9.1%)
	ICA	1 (4.6%)
Anterior versus posterior	Anterior	13 (59.1%)
Aneurysm laterality	Left	4 (18.2%)
	Right	4 (18.2%)
	BA or AComA	14 (63.6%)
Aneurysm type	Saccular	21 (63.6%)
	Dissecting	1 (4.6%)
	Blister	0 (0.0%)
	Fusiform	0 (0.0%)
Aneurysm neck – width (mm)		3.9 ± 1.7
Aneurysm sac – width (mm)		6.0 ± 4.3
Aneurysm sac – depth (mm)		6.2 ± 3.7
Dome-to-neck ratio		1.4 ± 1.0
Hunt & Hess grade	0	1 (4.6%)
	I	3 (13.6%)
	II	5 (22.7%)
	III	4 (18.9%)
	IV	4 (18.9%)
	V	5 (22.7%)
Fischer grade	I	7 (31.8%)
	II	4 (18.9%)
	III	3 (13.6%)
	IV	8 (36.4%)

AComA: anterior communicating artery; PComA: posterior communicating artery; MCA: middle cerebral artery; PICA: posterior inferior cerebellar artery; ICA: internal carotid artery; BA: basilar artery; SD: standard deviation.

### Clinical endpoints

The median mRS at discharge was 3 (2–5), indicating a moderate to moderately severe level of disability in the acute phase following aneurysm rupture and treatment. At discharge, 45.5% of patients had a favorable clinical outcome (mRS 0–2). Functional outcomes improved markedly during the early recovery phase, with a median mRS of 0 (0–2) at the 3-month follow-up with 81.8% of patients achieving mRS 0–2. This improvement was statistically significant, as confirmed by the Wilcoxon signed-rank test (*p* = 0.002). This positive trend continued over time: at the last documented follow-up, the median mRS further improved to 0 (0–1), with 90.9% of patients maintaining a favorable clinical outcome (mRS 0–2). The change from discharge to last follow-up was also statistically significant (Wilcoxon signed-rank test, *p* = 0.001). These findings underline a substantial and sustained functional recovery following SAC with the pEGASUS-HPC stent.

External ventricular drainage (EVD) was required in 15 patients (68.2%). The median time from aneurysm rupture to endovascular treatment was 0 days (IQR: 0–1), indicating early intervention in most cases. The average duration of hospitalization was 17.0 ± 10.1 days (Table 2).

**Table 2.** Procedural, device, and vessel characteristics, radiographic and clinical outcomes.

Parameters		*N* (%)/mean ± SD/median (IQR)
Number of devices	1 stent	16 (72.7%)
	2 stents (Y-stenting)	6 (27.3%)
Stent diameter		3.8 ± 0.5 mm
Stent length		22.7 ± 3.7 mm
Second device diameter (in Y-stenting cases)		3.7 ± 0.4 mm
Second device length (in Y-stenting cases)		20.8 ± 4.9 mm
Number of used coils		4 (1–11)
Proximal artery diameter		2.7 ± 1.1 mm
Distal artery diameter		1.9 ± 0.6 mm
Proximal second artery diameter (in Y-stenting cases)		2.3 ± 0.6 mm
Distal second artery diameter (in Y-stenting cases)		1.7 ± 0.6 mm
EVD insertion		15 (68.2%)
Days from aneurysm rupture to intervention		0 (0–1)
Days of hospitalization		17.0 ± 10.1
MRRC postintervention	Class I	19 (86.4%)
	Class II	0
	Class IIIa	3 (13.6%)
	Class IIIb	0
MRRC at 3-month follow-up	Class I	22 (100%)
	Class II	0
	Class IIIa	0
	Class IIIb	0
mRS at discharge		3 (2–5)
mRS at 3-month follow-up		0 (0–2)
mRS at last follow-up		0 (0–1)

EVD: external ventricular drain; IQR: interquartile range; SD: standard deviation; MRRC: modified Raymond-Roy occlusion classification; mRS: modified Rankin scale.

### Imaging and procedural endpoints

**Figure 1.**
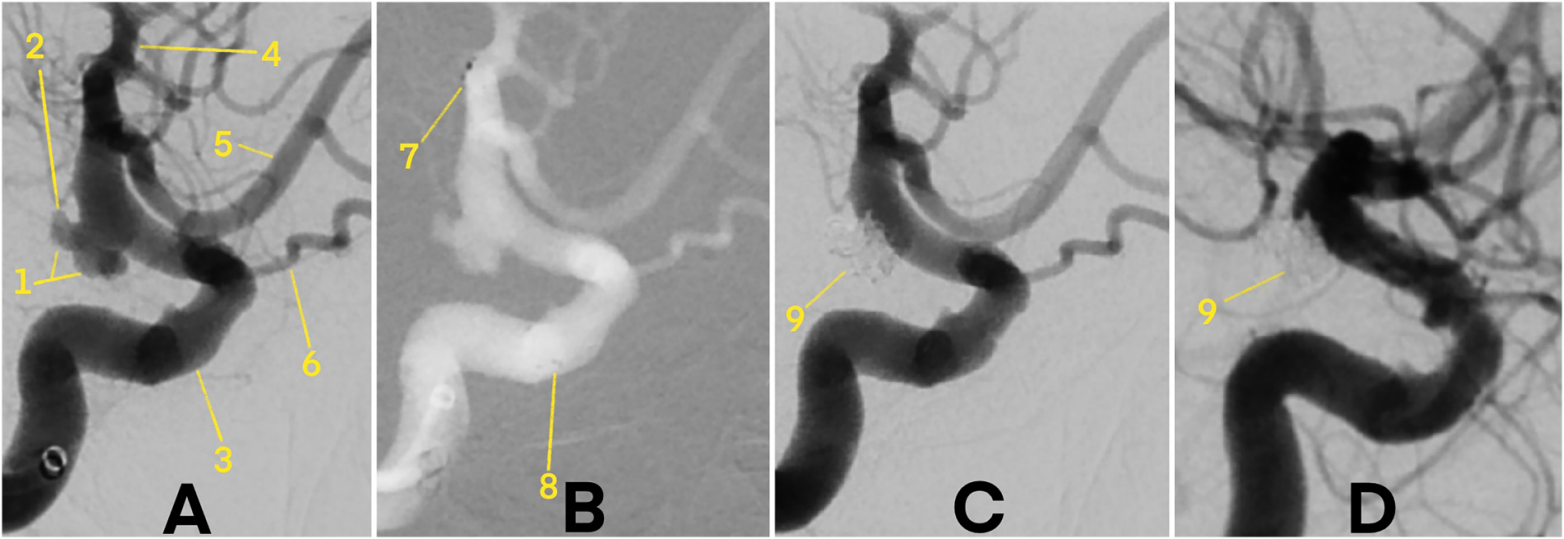
Digital subtraction angiography (DSA) demonstrating a wide-necked, ruptured aneurysm at the intradural terminal segment of the right internal carotid artery (ICA), treated with pEGASUS-HPC stent-assisted coiling. 1 – Ruptured ICA aneurysm. 2 – Presumed rupture site. 3 – ICA. 4 – Middle cerebral artery (MCA). 5 – Anterior cerebral artery (ACA). 6 – Ophthalmic artery. 7 – Distal stent markers. 8 – Proximal stent markers. 9 – Coiled aneurysm. (A) Pre-treatment angiogram showing vascular anatomy prior to stent and coil deployment. (B) Angiogram immediately after pEGASUS-HPC stent placement, with visible distal and proximal stent markers, but before coil embolization. (C) Post-coiling angiogram showing complete occlusion of the aneurysm sac with no procedural complications. (D) Follow-up DSA at 3 months confirming stable, complete aneurysm occlusion.

**Figure 2.**
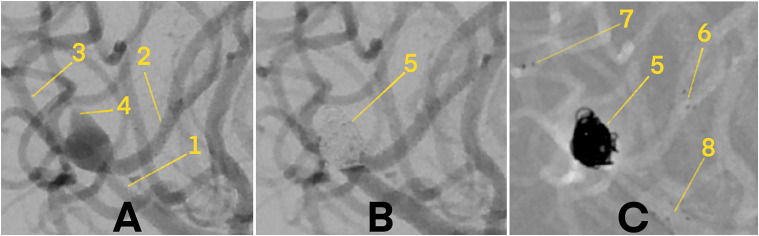
Digital subtraction angiography (DSA) of a wide-necked, ruptured aneurysm at the bifurcation of the middle cerebral artery (MCA), treated with stent-assisted coiling (SAC) using two pEGASUS-HPC stents in a Y-configuration. 1 – M1 segment of the MCA. 2 – Anterior M2 branch. 3 – Posterior M2 branch. 4 – Ruptured MCA aneurysm. 5 – Coiled aneurysm. 6 – Distal markers of the anterior pEGASUS-HPC stent. 7 – Distal markers of the posterior pEGASUS-HPC stent. 8 – Proximal markers of both pEGASUS-HPC stents. (A) Pre-treatment angiogram illustrating the vascular anatomy before stent and coil placement. (B) Final angiogram after SAC, demonstrating complete occlusion of the aneurysm sac without procedural complications. (C) Angiogram following deployment of both pEGASUS-HPC stents in Y-configuration and coil embolization, with clearly visible proximal and distal stent markers.

Immediate postprocedural complete occlusion (MRRC class I) was achieved in 19 patients (86.4%), while 3 patients (13.6%) had residual filling (class IIIa) (**Figures 1 and 2**). No patients showed class II or class IIIb occlusion. At the 3-month follow-up, angiographic imaging confirmed complete aneurysm occlusion (MRRC class I) in all 22 patients (100%).

A median of 4 coils (IQR: 1–11) was used to achieve stable aneurysm occlusion, depending on individual aneurysm characteristics. Device deployment was technically successful in all cases. A single pEGASUS-HPC stent was implanted in 16 patients (72.7%), while 6 patients (27.3%) required dual-stent implantation in a Y-configuration. The mean diameter and length of the primary stent were 3.8 ± 0.5 mm and 22.7 ± 3.7 mm, respectively. In Y-stenting cases, the second stent measured 3.7 ± 0.4 mm in diameter and 20.8 ± 4.9 mm in length (Table 2).

### Comparison between survived and deceased patients

There were no statistically significant differences between survivors and deceased patients in terms of age, gender, EVD insertion, technical aspects (single stent vs Y-stenting), or treatment delay. However, all deceased patients (3/3, 100%) had aneurysms located in the BA, compared to 4 of 19 survivors (21.1%), suggesting a trend toward worse outcomes in this anatomical subgroup (*p* = 0.051). Aneurysm neck width was also greater in non-survivors (4.8 ± 1.4 mm vs 3.6 ± 1.7 mm), although this difference did not reach statistical significance (*p* = 0.149). Most notably, all patients who died (3/3, 100%) presented with Hunt and Hess grade V at admission. Of the five patients with grade V in the cohort, 60% (3/5) died, whereas no deaths occurred among patients with grades 0–IV. This strong association between poor clinical grade and mortality was statistically significant (*p* = 0.0048). Functional outcomes at discharge also differed clearly between groups: deceased patients had a median mRS of 5 (4–5), whereas survivors had a median mRS of 3 (2–5). These results suggest that Hunt and Hess grade V was the strongest predictor of mortality, outweighing anatomical and procedural characteristics, while BA location also showed a potential association with worse outcome.

### Observed complications

Complications occurred in 2 patients (9.1%), while the remaining 20 patients (90.9%) experienced no procedural or postprocedural adverse events. One patient (4.6%) developed transient in-stent thrombosis, detected on the first DSA control run performed immediately after completion of coil deployment. This indicates that the thrombosis likely formed between stent placement and the end of coiling. The event resolved spontaneously following the administration of an intravenous tirofiban bolus, with no clinical sequelae. In another patient (4.6%), progression of SAH was observed on early postinterventional imaging. As the preprocedural CT scan showed no increase in hemorrhage, the re-hemorrhage most likely occurred during the intervention. Notably, no retreatment was required in any case.

### Y-Stenting subgroup analysis

Of the 22 patients included in this study, 6 (27.3%) underwent Y-stenting. In this subgroup, no procedural or postprocedural complications were observed. All six patients achieved a favorable clinical outcome (mRS 0–2) at the last documented follow-up. Moreover, complete aneurysm occlusion (MRRC I) was achieved in all Y-stenting cases. These results were comparable to those in the single-stent group, suggesting that Y-stenting using the pEGASUS-HPC stent is both safe and technically feasible in appropriately selected patients.

## Discussion

This multicenter retrospective study evaluated the clinical and imaging outcomes of SAC using the pEGASUS-HPC stent in patients with acutely ruptured wide-necked intracranial aneurysms. The results demonstrate high rates of procedural success, low complication rates, and encouraging functional outcomes, suggesting the device's feasibility and potential safety in this challenging clinical setting.

The immediate postprocedural complete occlusion rate (MRRC class I) was 86.4%, increasing to 100% at the 3-month follow-up, indicating progressive thrombosis and durable aneurysm exclusion. These results align with prior reports by Boxberg et al., who described complete occlusion in 83.3% of cases and 90.9% at follow-up using the same device.^14^ Our findings also build upon those by Pielenz et al., who demonstrated successful deployment of the pEGASUS-HPC stent in both elective and emergency settings, emphasizing its versatility and safety.^11^

Our findings compare favorably with a recent meta-analysis by Bsat et al., which reported a total complication rate of 20.8%, a thromboembolic rate of 9.1%, a hemorrhagic rate of 8.7%, and a mortality rate of 7.8% in patients treated with other non-coated stent systems, including Neuroform, Enterprise, Solitaire, LEO, Acclino, LVIS, and balloon-expandable stents. In contrast, our cohort demonstrated a lower total complication rate (9.1%), no hemorrhagic events, and a higher rate of favorable clinical outcome (mRS 0–2: 90.9%). These differences may in part be attributable to the antithrombogenic coating of the pEGASUS-HPC stent.^16^

Patients exhibited a clear and sustained improvement in functional status following treatment. The median mRS decreased from 3 (2–5) at discharge to 0 (0–2) at the 3-month follow-up, reflecting early recovery in most cases. This favorable trend persisted, with a further narrowing of the IQR to 0 (0–1) at last follow-up, underscoring the durability of clinical outcomes.

Complications were infrequent, occurring in only 2 patients (9.1%). One was device-related, involving a transient in-stent thrombosis that resolved spontaneously following tirofiban administration. The other was a radiologically detected progression of SAH on post-interventional CT imaging. Both events were managed conservatively and did not result in clinical deterioration. Although these results are encouraging, they should be interpreted with caution given the limited cohort size and lack of a control group.

In 6 patients (27.3%), Y-stenting was required, emphasizing the flexibility of the pEGASUS-HPC system to accommodate complex anatomies. The median time from rupture to intervention was 0 (0–1) days, demonstrating the feasibility of early SAC even in acutely ruptured aneurysms, provided adequate antiplatelet management is ensured.

A practical advantage observed in this cohort was the ability to deploy the stent via the same microcatheter used for coiling (0.0165”), which avoided the need for microcatheter exchange, as described in previous studies using the pEGASUS-HPC stent.^17^ This feature may contribute to procedural safety by reducing manipulation within the parent artery and minimizing delays during the intervention—particularly relevant in hemodynamically unstable or neurologically fragile SAH patients.

Although long-term durability beyond one year could not be assessed in this study, prior reports on laser-cut stents such as the Neuroform Atlas and Enterprise have demonstrated stable occlusion and low recurrence rates over extended follow-up periods.^16^ Whether comparable durability can be expected from the pEGASUS-HPC stent remains to be confirmed in future longitudinal studies with imaging beyond 12 months.

Three of 22 patients (13.6%) died during the clinical course, all of whom had Hunt and Hess grade V and BA aneurysms. This highlights the strong influence of initial clinical status on prognosis in the setting of acute SAH. While age, gender, treatment timing, and procedural technique did not differ significantly between survivors and non-survivors, high-grade hemorrhage at admission was significantly associated with mortality.

In addition to poor clinical grade, the BA location may have contributed to the fatal outcomes. Although no technical or access-related difficulties occurred in our cohort, aneurysms in this region lie near the brainstem and may cause secondary injury via compression or mass effect—particularly in the setting of raised intracranial pressure or hydrocephalus. These factors may amplify the impact of poor neurological status at presentation and should be considered during treatment planning and prognostic assessment.

### Clinical implications and future directions

Our findings suggest that the pEGASUS-HPC stent is a promising tool for the treatment of acutely ruptured wide-necked intracranial aneurysms, offering high rates of aneurysm occlusion with a low complication profile. The observed functional recovery in most patients highlights the potential benefit of early, technically precise intervention using surface-modified stents in the acute setting. Nevertheless, clinical outcomes were strongly influenced by initial neurological status, emphasizing the need for timely patient triage and careful risk stratification. Future research should include prospective multicenter registries and randomized controlled trials comparing surface-modified (HPC-coated) versus uncoated stents to provide high-level evidence regarding safety, efficacy, and optimal antiplatelet strategies. Such studies could clarify whether the antithrombogenic coating results in clinically meaningful advantages in terms of thromboembolic or hemorrhagic risk, especially in patients with SAH. Furthermore, the role of shortened or tailored antiplatelet regimens in this vulnerable population warrants investigation.

### Limitations

This study is subject to several limitations. Its retrospective design introduces potential biases, such as incomplete data capture and selection effects. The relatively small sample size limits both statistical power and the generalizability of the results. Furthermore, the lack of a comparison group treated with other stent systems restricts conclusions regarding the relative efficacy of the pEGASUS-HPC stent. Although imaging follow-up was uniformly conducted up to one year, long-term outcomes such as aneurysm recurrence and delayed complications remain unassessed.

### Conclusion

SAC using the pEGASUS-HPC stent appears to be a safe and effective strategy for treating acutely ruptured wide-necked intracranial aneurysms. In this multicenter cohort, high rates of complete occlusion were achieved with low complication rates and no need for retreatment. Most patients experienced substantial and sustained recovery, particularly when treated early and in the absence of poor-grade clinical presentation. Although these results are encouraging, they should be interpreted with caution given the limited sample size and lack of a control group. These findings underscore the clinical value of surface-modified stents in the acute SAH setting and highlight the need for prospective, comparative studies to validate these findings.

## Abbreviations

ACA: anterior cerebral artery
AComA: anterior communicating artery
ASA: acetylsalicylic acid
BA: basilar artery
CTA: computed tomography angiography
CTP: computed tomography perfusion
DSA: digital subtraction angiography
EVD: external ventricular drain
HPC: hydrophilic polymer coating
ica: internal carotid artery
IQR: interquartile range
ISAT: International Subarachnoid Aneurysm Trial
mRS: modified Rankin scale
MRRC: modified Raymond-Roy classification
MRI: magnetic resonance imaging
MCA: middle cerebral artery
PComA: posterior communicating artery
PICA: posterior inferior cerebellar artery
SAH: subarachnoid hemorrhage
SAC: stent-assisted coiling
SD: standard deviation
TOF: time-of-flight (MR angiography)
